# Supplementary material for: Reconciling Mining with the Conservation of Cave Biodiversity: A Quantitative Baseline to Help Establish Conservation Priorities
Source: PLoS One. 2016 Dec 20;11(12):e0168348. doi: 10.1371/journal.pone.0168348 (PMC5173368; doi:10.1371/journal.pone.0168348)
Supplement: S1 Dataset — (ZIP) [file pone.0168348.s002.zip › Taxa/Serra Norte/SN_2013/N4WS/Lista N4WS-67.pdf]

CAVIDADE N4WS-0067

Classe	Ordem	Fam/Outros	Gên/Outros	Espécie	S-U	U-S	Zon.
Annelida	Oligochaeta			sp.2	3	3	E/P/A
Arachnida	Acari	(Oribatida)		sp.1		X	P
Arachnida	Acari	(Parasitengonina)		sp.2	X		A
Arachnida	Acari	(Parasitengonina)		sp.3		X	P
Arachnida	Acari	Argasidae	*Ornithodoros*	sp.1		X	P
Arachnida	Acari	Rhagidiidae		sp.1	X		P
Arachnida	Acari	Tydeidae		jovem		X	A
Arachnida	Amblypygi	Charinidae	*Charinus*	sp.1	2		E
Arachnida	Araneae	Araneidae	*Mangora*	sp.1	1		E
Arachnida	Araneae	Caponiidae		sp.1	X	X	A
Arachnida	Araneae	Ctenidae		jovem	2		E/P
Arachnida	Araneae	Ochyroceratidae	*Ochyrocera*	sp.1	X	X	P/A
Arachnida	Araneae	Ochyroceratidae	*Speocera*	sp.1		X	E
Arachnida	Araneae	Ochyroceratidae		sp.n.1	X	X	A
Arachnida	Araneae	Oonopidae		jovem	X		A
Arachnida	Araneae	Pholcidae	*Leptopholcus*	sp.1	X	X	P
Arachnida	Araneae	Scytodidae	*Scytodes*	*eleonorae*	1	1	E/P
Arachnida	Araneae	Segestriidae		jovem		X	P
Arachnida	Araneae	Theraphosidae	aff. *Holothele*	sp.1	8	2	E
Arachnida	Araneae	Theridiidae	*Achaearanea*	sp.1	X	X	E
Arachnida	Opiliones	Escadabiidae		jovem		X	E
Arachnida	Pseudoscorpiones	Bochicidae		sp.1	X		A
Arachnida	Pseudoscorpiones	Chernetidae	*Spelaeochernes*	sp.1	X	X	P
Arachnida	Pseudoscorpiones	Chthoniidae	*Pseudochthonius*	sp.1		X	E/P
Arachnida	Pseudoscorpiones	Chthoniidae	*Pseudochthonius*	sp.3	X		A
Arachnida	Pseudoscorpiones	Chthoniidae		sp.4	X		P
Arachnida	Schizomida	Hubbardiidae	aff. *Rowlandius*	sp.1	X	X	E/P
Chilopoda	Scutigeromorpha	Pselliodidae		jovem		2	A
Diplopoda	Polydesmida	Pyrgodesmidae		sp.1	X		A
Diplopoda	Polyxenida	Hypogexenidae		sp.1		X	A
Entognatha	Collembola	Paronellidae		sp.1		X	P
Entognatha	Collembola	Paronellidae		sp.4	X	X	A
Entognatha	Collembola	Symphypleona		sp.1	X		P
Entognatha	Diplura	Campodeidae		sp.1		X	P
Insecta	Coleoptera	Carabidae		jovem	X		E
Insecta	Coleoptera	Curculionidae	Scolytinae	sp.2	X		P
Insecta	Coleoptera	Dytiscidae	*Copelatus*	*cessaima*	X		A
Insecta	Coleoptera	Dytiscidae		jovem		X	E
Insecta	Coleoptera	Dytiscidae		sp.1	X	X	E/P/A
Insecta	Coleoptera	Dytiscidae		sp.2		X	E
Insecta	Coleoptera	Elminthidae		jovem	X		A
Insecta	Coleoptera	Staphylinidae		sp.35	X		E
Insecta	Coleoptera	Tenebrionidae		jovem		X	E/P
Insecta	Diptera	Ceratopogonidae		sp.	X	X	A
Insecta	Diptera	Culicidae		jovem	X		P
Insecta	Diptera	Phoridae	Phorinae	sp.		X	A
Insecta	Diptera	Psychodidae	*Pintomyia*	*gruta*		X	P
Insecta	Diptera	Psychodidae	*Sciopemyia*	*sordellii*	X	X	E/P
Insecta	Diptera	Sarcophagidae		jovem	X		E

Insecta	Diptera	Sphaeroceridae		sp.	X		E
Insecta	Heteroptera	Ceratocombidae		sp.2	X		E
Insecta	Heteroptera	Reduviidae	Emesinae	jovem		1	P
Insecta	Heteroptera	Reduviidae	Reduviinae	jovem	1		P
Insecta	Heteroptera	Schizopteridae	Schizopterinae	sp.1	X	X	P/A
Insecta	Heteroptera	Veliidae	*Paravelia*	sp.1	X	X	E/P
Insecta	Heteroptera	Veliidae	*Paravelia*	sp.2	X	X	A
Insecta	Hymenoptera	Formicidae	*Camponotus*	*atriceps*		2	P
Insecta	Hymenoptera	Formicidae	*Camponotus*	sp.1	X		P
Insecta	Hymenoptera	Formicidae	*Pachycondyla*	*striata*	2	1	E/P
Insecta	Hymenoptera	Formicidae	*Strumigenys*	sp.1		X	A
Insecta	Lepidoptera	Noctuoidea		sp.1	1	13	E/P
Insecta	Lepidoptera	Noctuoidea		sp.12	1		E
Insecta	Lepidoptera	Tineoidea		sp.6	X	X	P
Insecta	Orthoptera	Phalangopsidae	*Paraclodes*	sp.	33		E
Insecta	Orthoptera	Phalangopsidae	*Phalangopsis*	sp.	96	86	P/A
Insecta	Psocoptera	Psocomorpha		jovem	X	X	E
Insecta	Psocoptera	Psyllipsocidae		jovem		X	P
Insecta	Thysanura	Nicoletiidae		sp.1	X	X	E
Gastropoda	Pulmonata	Systrophiidae	*Happia*	sp.	X		E
Malacostraca	Isopoda	Scleropactidae	*Circoniscus*	*buckupi*	X	X	P/A
Amphibia	Anura	Leptodactylidae	*Leptodactylus*	cf. *vastus*	1		E
Amphibia	Anura	Strabomantidae	*Pristimantis*	cf. *fenestratus*	2	1	E/P
Mammalia	Chiroptera	Emballonuridae	*Peropteryx*	sp.	10	8	
Mammalia	Chiroptera	Natalidae	*Natalus*	*stramineus*	5	1	
Mammalia	Chiroptera	Phyllostomidae	*Carollia*	*perspicillata*		1	
